# Pheromone-Mediated Mating Disruption as Management Option for *Cydia* spp. in Chestnut Orchard

**DOI:** 10.3390/insects12100905

**Published:** 2021-10-05

**Authors:** Chiara Ferracini, Cristina Pogolotti, Franco Rama, Giada Lentini, Valerio Saitta, Pierangelo Mereghetti, Paolo Mancardi, Alberto Alma

**Affiliations:** 1 Department of Agricultural, Forest and Food Sciences (DISAFA), University of Torino, Largo Paolo Braccini 2, 10095 Grugliasco, Italy; cristina.pogolotti@unito.it (C.P.); giada.lentini@unito.it (G.L.); valerio.saitta@unito.it (V.S.); paolo.mancardi@unito.it (P.M.); alberto.alma@unito.it (A.A.); 2 Isagro S.p.A.—Biological Products Unit, Via Fauser, 28, 28100 Novara, Italy; franco.rama24@gmail.com (F.R.); pmereghetti@isagro.com (P.M.)

**Keywords:** *Cydia fagiglandana*, *Cydia splendana*, Ecodian^®^ CT, Lepidoptera, *Castanea sativa*, integrated pest management

## Abstract

**Simple Summary:**

Investigations were conducted to evaluate the effectiveness of mating disruption (MD) to control chestnut tortrix moths. Surveys were performed at four sites in northern Italy in 2019–2020. MD was carried out using the pheromone dispenser Ecodian^®^ CT. The total number of trapped males was significantly lower in MD plots than in control ones for all sites and years. Trap catch suppression in MD plots averaged 89.5% and 93.8% for *Cydia fagiglandana* and 57.4% and 81% for *Cydia splendana* in 2019 and 2020, respectively. A reduction of about 71% of larval infestation of chestnut fruits was only recorded in the MD plot at one site in 2019. Although the reduction in male catches in MD plots was observed, this research highlighted the need to perform further studies to investigate the effect of MD in reducing larval damage of chestnut fruits.

**Abstract:**

(1) Background: Pheromone-based devices are successfully used to control insect pests in agriculture. (2) Methods: Investigations were conducted to evaluate the effectiveness of mating disruption (MD) to control the chestnut tortrix moths, *Cydia fagiglandana* and *Cydia splendana*. Surveys were performed in northern Italy in 2019–2020. MD was carried out using the pheromone dispenser Ecodian^®^ CT. The effectiveness of MD was assessed by recording male adult catches in pheromone-baited sticky traps and larvae in chestnut fruits, comparing MD and control plots. (3) Results: The total number of trapped males was significantly lower in MD plots than in control ones, for all sites and years. Trap catch suppression in MD plots averaged 89.5% and 93.8% for *C. fagiglandana* and 57.4% and 81% for *C. splendana* in 2019 and 2020, respectively. The larval infestation rate in fruits did not vary between plots except for one site where a reduction of about 71% in the MD plot was recorded in 2019. (4) Conclusions: Low catches in MD plots turned out to be a good measure of the effectiveness of communication disruption, but no satisfactory data have been obtained regarding fruit infestation, highlighting how the reduction of male catches cannot always be considered as a reliable indicator of successful control. Specific investigations about background population density, dispersal and mating/oviposition behavior are thus essential for a viable management strategy.

## 1. Introduction

In the last decades, the development of effective and environmentally sustainable pest management strategies, reducing pesticide use in pest control, has been strongly encouraged, aiming to obtain residue-free foods and fewer negative impacts of chemicals on human health and the environment [1]. The potential of using synthetic pheromones for the control of moths and other pests has long been recognized [2,3]. In particular, mating disruption (MD) technology uses synthetically produced chemicals in large amounts to confuse males and limit their ability to locate calling females, thus reducing pest mating and preventing crop damage [4,5,6]. MD has been one of the most successfully used strategies for controlling various pests with over 800,000 hectares treated worldwide [7]. Non-target effects are reported to be rare due to the high specificity of pheromones to which only the target species respond [8], and neither the target organism nor other species are killed [9]. Moreover, the MD technique can decrease the risk of resistance to pesticides and is compatible with IPM and biological control strategies, making mating disruption a key tool regarding the suppression of insect pests in a variety of managed ecosystems [5,10].

Pheromone-based devices have been successfully used to control insect pests in agriculture, including orchards, vineyards, annual vegetables and fiber crops [8]. A wide variety of pheromone dispensers are commercially available, such as dispensers spraying microencapsulated pheromones such as insecticides, hand-applied dispensers with a reservoir and permeable membrane to regulate release, hollow fibers, wires and twist-tie ropes [8]. In the literature, hundreds of female sex pheromones are reported, in particular for lepidopteran species [10], and they are used for MD for more than 20 insect species worldwide [11]. As reported by Wijayaratne and Burks [12], MD has been developed for lepidopteran pests of stored products, such as for *Plodia interpunctella* (Hübner), as well as *Cadra cautella* (Walker) and *Ephestia kuehniella* Zeller (Lepidoptera: Pyralidae). Moreover, pheromone dispenser applications have successfully controlled several lepidopteran pests on crops and in the forestry environment, as in the case of *Tuta absoluta* (Meyrick) (Lepidoptera: Gelechiidae) [13] and the gypsy moth *Lymantria dispar* (L.) (Lepidoptera: Erebidae) [14]. Various formulations have been successfully adopted to control lepidopteran tortricid, such as the codling moth *Cydia pomonella* (L.) and the Oriental fruit moth *Grapholita molesta* (Busck) (Lepidoptera: Tortricidae) in apple and stone fruit orchards [15,16,17,18,19]. Applications have been reported to control the European grapevine moth *Lobesia botrana* (Denis and Schiffermüller) (Lepidoptera: Tortricidae) in vineyards as well, even if only at high population density [20]. Furthermore, applications to control *Cydia fagiglandana* (Zeller) and *C. splendana* (Hübner) (Lepidoptera: Tortricidae) have been investigated [21,22]. Among native pests, these tortricid species are considered as the key moth pests of chestnut in Europe, causing fruit losses of up to 70% in harvested fruits depending on the year and plantation [23]. Pheromone blends have been actively investigated for their control, and the use of sexual pheromones in specific control programs such as MD, in conjunction with cultural techniques, are considered as the most efficient measures of control for these species [24,25].

In the present paper, we report a 2-year study that was carried out to investigate the effectiveness of MD to control the chestnut tortrix moths, *C. fagiglandana* and *C. splendana*, in northern Italy. The main objective was to assess the effectiveness of MD with wire dispensers for *Cydia* spp. moths which we evaluated through ratios of trap catch suppression (reduction in male catches in the pheromone traps) and damage in chestnut fruits, comparing MD plots vs. untreated ones.

## 2. Materials and Methods

### 2.1. Survey Sites

This research was carried out in a two-year period (2019–2020) in sweet chestnut orchards (*Castanea sativa* Miller) located in four sites in four northern Italian regions, namely Emilia Romagna, Liguria, Piedmont and Tuscany. The survey sites were characterized by managed chestnut orchards. Trees were approximately 30 years old, 15–20 m in height, planted at 8 m distance along the row and 8 m distance between rows. Tree density was about 120 trees/hectare. For each site, one chestnut orchard of about 1 ha in size was chosen to perform MD, and sticky traps were used to monitor the presence of the target species. Plots similar in tree size, height and variety were chosen as control. To avoid interference, untreated control plots were chosen at least 2 km far away from the MD ones. Preliminary surveys were performed in previous years to choose sites with similar damage by *C. fagiglandana* and *C. splendana* larvae (Ferracini C, unpublished). No insecticide treatment was ever applied in the plots. All information concerning the surveyed sites is given in the Supplementary Table S1.

### 2.2. Monitoring Traps

The population dynamics of *C. fagiglandana* and *C. splendana* were investigated using sexual pheromone lures (codlemone acetate: (E,E)-8,10-dodecadien-1-yl acetate; codlemone: (E,E)-8,10-dodecadien-1-ol) and sticky traps (Traptest^®^, Isagro S.p.A., Novara, Italy). The amounts of synthetic pheromone loaded onto each septum cannot be reported as these are trade secrets of the manufacturer. Three traps per ha per species were hung on chestnut branches at 5–6 m in height in the outer surface of tree canopy. A randomized complete block design was used according to the methods described by Ferracini et al. [26]. Pheromone lures were replaced every four weeks according to the manufacturer’s recommendations, whereas traps were inspected weekly. At each inspection, the sticky surface of the trap was removed and replaced with a new one. All the sticky surfaces removed were taken to the laboratory to count and identify adult moths. All other insects different from the target moths were discarded.

### 2.3. Mating Disruption

MD was performed using the pheromone dispenser Ecodian^®^ CT (Isagro S.p.A., Novara, Italy), consisting of a wire of paper impregnated with pheromone components of *C. fagiglandana* and *C. splendana* (codlemone acetate and codlemone) and coated with a layer of biodegradable material based on Mater-Bi^®^ (cornstarch and thermoplastic polymers). Ecodian^®^ CT wire, 3 mm in diameter, was loaded with 18.75 g/ha of codlemone acetate and 6.25 g/ha of codlemone. In each treated chestnut orchard, segments of wire (5 m in length) were vertically placed on each tree with the aid of a telescopic rod. Each wire ended with a hook for easy hanging on the branch (Supplementary Figure S1). In total, 900 m of wire per ha were placed evenly in each MD chestnut orchard before the start of adult flights. Pheromone application was performed between the end of June and the beginning of July, avoiding rainy days. The application period was chosen in relation to the monitoring data already available for the target species in the surveyed sites [26]. Since borders of pheromone-treated orchards can be susceptible to different levels of pheromone concentrations [27,28], Ecodian^®^ CT wires were applied on all trees surrounding the chestnut orchards within 10 m outside the perimeter of the plot. The efficacy of the MD was assessed by comparing the male adult catches in the pheromone-baited traps, comparing MD vs. control plots.

### 2.4. Ecodian^®^ CT Pheromone Release Rate

To measure the pheromone release rate, 9 pieces of Ecodian^®^ CT about 2 m long were placed in a chestnut orchard in the Piedmont region, tied on the main branches. Each piece was sealed at both ends with insulating tape. One piece was removed every 10 days, wrapped in foil, taken to the laboratory, and placed in the freezer at −25 °C and then analyzed.

### 2.5. GC Analysis

The analyses were performed by gas-chromatography with internal standard. GC-FID analyses were carried out on a Focus GC (Thermo Finnigan) instrument equipped with a SUPELCO Equity-5 wide bore Capillary Column (30 m; 0.53 mm I.D.; DF = 1.5 μm). The GC conditions were as follow: the injector temperature at 250 °C, oven thermal program from 100 °C to 280 °C with gradient of 15 °C/min, then hold for 5 min at 280 °C; using helium as carrier (2.5 mL/min). The detector was a flame ionization detector (FID) kept at 300 °C.

Samples were prepared taking 30 cm of pheromone impregnated thread exactly measured, cut in small pieces about 1–2 cm in length and put in a glass bottle with 20 mL of internal standard solution (1-hexadecanol 0.5 mg/mL in acetone). Samples were left overnight, then an aliquot was taken and analyzed (injection volume 1 μL). The analysis was replicated twice. The compounds were identified by comparison with authentic standards. Results were expressed as percent of residual pheromones amount.

### 2.6. Fruit Collection

A sample of at least 900 chestnut husks was randomly hand-picked from the ground three times during the fruit fall, according to the method described in Ferracini et al. [26]. Nuts were visually inspected after collection for the presence of exit holes, then stored in cardboard boxes in outdoor conditions and checked daily to record the number of newly emerged larvae. Only tortricid larvae were considered and any larva belonging to the chestnut weevil *Curculio elephas* Gyllenhaal (Coleoptera: Curculionidae) and/or any emergence hole with a typical diameter attributable to *C. elephas* (>2.5 mm) was discarded. Fruit collection was carried out in Villar Focchiardo, Montese and Badia del Borgo in 2019 and in Villar Focchiardo and Montese in 2020. Stored fruits were checked daily until no emergence was recorded for three consecutive weeks.

### 2.7. Identification of Specimens

All male adults caught in the sticky traps were morphologically identified by observing the male genital structures as described in Ferracini et al. [26]. The larvae obtained from chestnut fruits were identified based on morphological traits and/or compared with voucher specimens deposited at the DISAFA-Entomology laboratory. Doubtful adult and larvae specimens were submitted for DNA extraction according to the method described by Asghar [29] and then sequenced for the cytochrome oxidase I (COI) gene following Hajibabaei et al. [30]. The sequences were compared with those in the National Center for Biotechnology Information sequence database.

### 2.8. Statistical Analysis

The field trials were conducted using a randomized complete block design and data were subjected to analysis of variance (ANOVA). All data were first tested for homogeneity of variance (Levene’s test) and log transformed to stabilize variances and normalize the data. The number of males captured in monitoring traps and the damage detected in chestnut fruits in MD and control plots for all surveyed sites and years were used as a replicate and compared by analysis of variance with Tukey’s test for multiple comparisons. Statistical significance was set at *p* < 0.05. All analyses were performed using SPSS version 22.0 (SPSS, Chicago, IL, USA).

## 3. Results

### 3.1. Trap Captures

**Figure 1:**
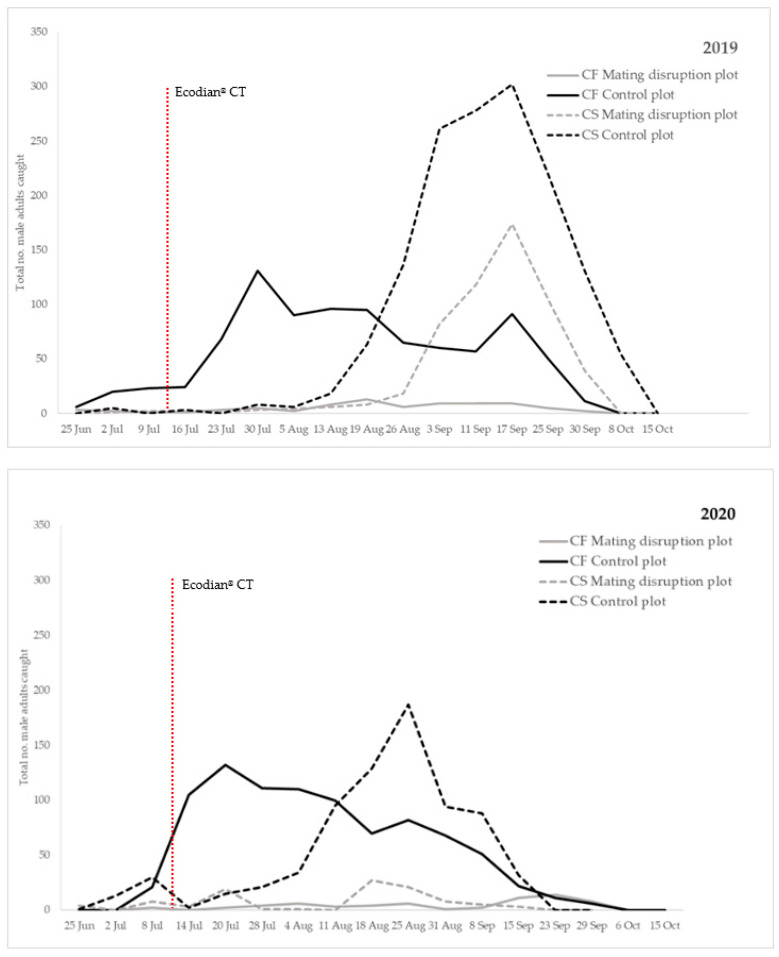
Total number of male adults/per week of *Cydia fagiglandana* (solid line) and *Cydia splendana* (dashed line) collected with pheromone-baited traps in all the surveyed sites, in plots where mating disruption (MD) (grey lines) was applied compared to control plots (black lines) in 2019 (**top**) and 2020 (**bottom**). The vertical dotted line corresponds to the Ecodian^®^ CT mating disruption application.

The seasonal flight activity, reported as the total number of male moths captured by traps for all species, sites and years, considering MD and control plots, is given in Figure 1.

Both investigated species were recorded in all surveyed sites, exhibiting a similar temporal pattern in both years, with small differences among the sites. The first record of *C. fagiglandana* occurred between 16 June and 25 June and lasted until late September, while *C. splendana* started the flight period later, being recorded between 16 July and 28 July until the end of October. On average, *C. fagiglandana* represented 74% of all the individuals found and, among the non-target species, *Cydia ilipulana* (Walsingham) and the pea moth *Cydia nigricana* F. (Lepidoptera: Tortricidae) were the most abundant.

*C. splendana* accounted for 86% and the non-target species, *C. ilipulana,* was the most abundant one, collected mostly in Piedmont region (Villar Focchiardo site). In 2019, MD and control plots showed the same period as flight peak, and capture data showed that the flight peaks of *C. fagiglandana* and *C. splendana* occurred on average in mid-August (4.0 ± 0.96 males/trap in 2019) and mid-September (46 ± 5.10 males/trap in 2019), respectively. The number of trapped males was significantly lower (t = 17.947; df = 1, 3; *p* = 0.01) in MD plots (53 males for *C. fagiglandana* in 2019) than in control ones (506 males for *C. fagiglandana* in 2019). Similar values were recorded regarding *C. splendana*, being the trapped males in MD plots (337 males for *C. splendana*) significantly lower (t = 24.301; df = 1, 3; *p* = 0.01) than in control ones (792 males for *C. splendana*) in all sites.

**Figure 2:**
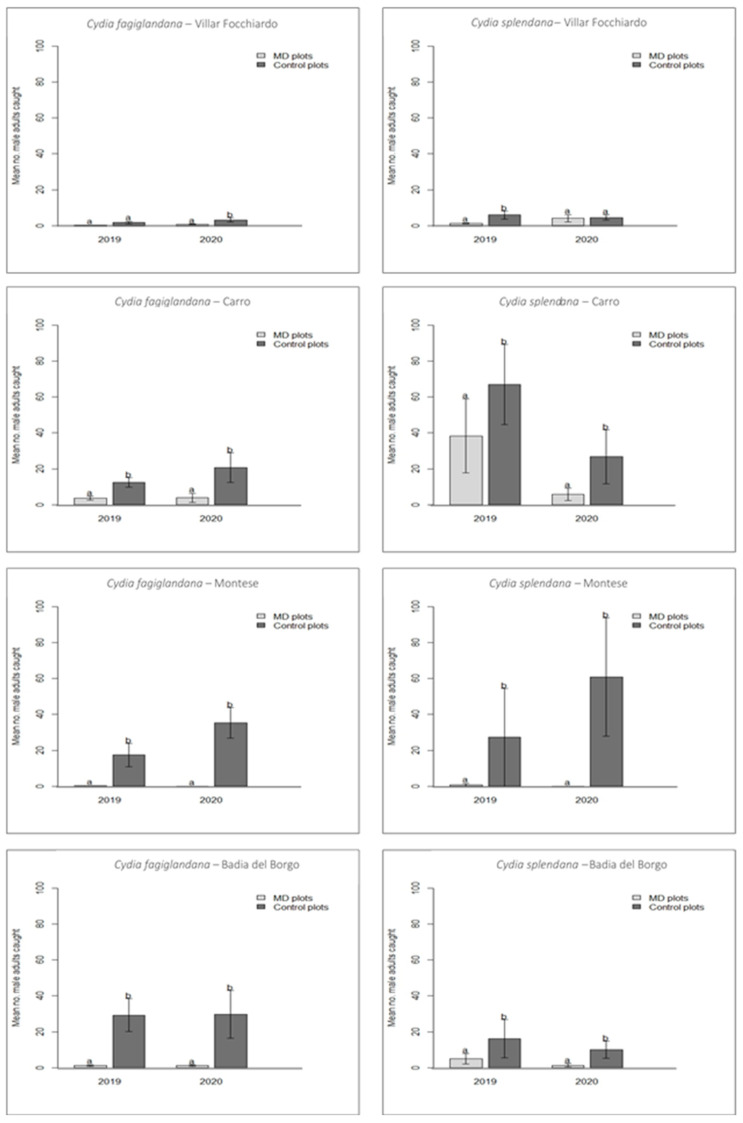
Average number (±SEM) of male adults of *Cydia fagiglandana* (**left**) and *Cydia splendana* (**right**) collected with pheromone-baited traps, in plots where mating disruption (grey histograms) was applied compared to control plots (black histograms) in 2019 and 2020. Bars with different letters are significantly different, according to the paired *t*-test (*p* < 0.05).

In 2020, capture data from the control plots showed that the flight peaks of *C. fagiglandana* and *C. splendana* occurred on average in late July (14.0 ± 1.33 males/trap in 2020) and late August (22 ± 7.30 males/trap in 2020), respectively. The population density of both target species was recorded slightly earlier in the season than 2019. Similarly to 2019, trap captures in MD plots were significantly higher than in control plots. The number of trapped males was significantly higher (t = 31.281; df = 1, 3; *p* = 0.01) in MD plots (36 males) than in control ones (576 males) for *C. fagiglandana*. Similar values were recorded with regard to *C. splendana*, being the trapped males in MD plots (83 males) significantly lower (t = 19.509; df = 1, 3; *p* = 0.01) than in control ones (437 males) in all sites. The average number (± SEM) of male adults of both species collected in MD and control plots in 2020 is reported in Figure 2.

Male trap catch suppression in MD plots averaged 89.5% and 93.8% for *C. fagiglandana*, and 57.4% and 81% for *C. splendana* in 2019 and 2020, respectively.

### 3.2. GC Analysis

**Figure 3:**
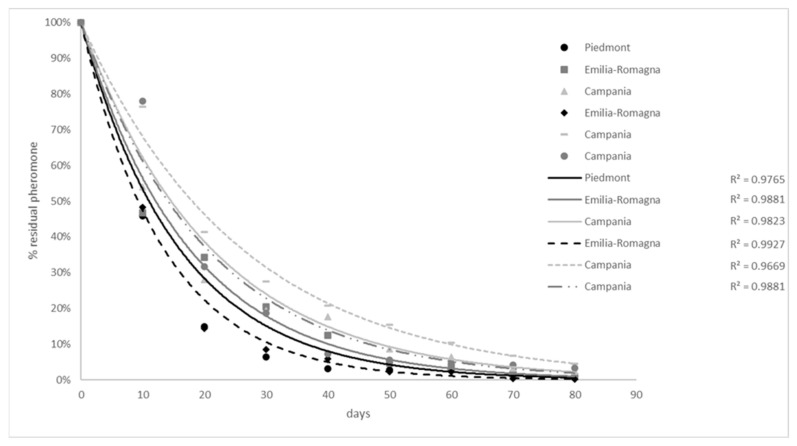
Release curve of Ecodian^®^ CT, showing the percentage of residual pheromone tested in Piedmont region (black solid line). The curve is compared to previous data recorded in different sites in Emilia-Romagna (dark grey solid line and black dotted line) and Campania regions (light grey solid line, light dotted line and dark grey dotted line).

The release curve of the pheromone blend, showing the percentage of residual pheromone in the thread, is reported in Figure 3. This curve is compared to previous data obtained following the same protocol described in the Section 2.4 and Section 2.5 in Emilia-Romagna and Campania regions (Mereghetti P and Rama F, unpublished).

### 3.3. Fruit Infestation

Regarding infested chestnut fruits, 89% of the larvae recorded was identified as *C. fagiglandana* and the remaining 11% was identified as *C. splendana*. In three sites out of five, the data for the proportion of larval damage recorded per site was lower in MD than control plot. However, no statistical differences were found when comparing these sites (2019: t = 2.57, df = 2, *p* = 4.17; 2020: t = 4.77, df = 1, *p* = 0.10), except for the Montese site in 2019 (Table 1).

## 4. Discussion

All the investigated species were recorded in all surveyed sites and in both years (2019–2020). The two tortrix species overlapped during the season, exhibiting a similar temporal pattern in different sites and years to previous investigations already carried out in northern Italy [26,31,32,33]. On average, *C. fagiglandana* and *C. splendana* represented 84% and 96% of all the individuals found in 2019–2020.

Although pheromones are species specific, it has often been observed that several non-target insects may be trapped owing to attractive pheromone blends. Recently, investigations carried out in the same surveyed sites highlighted how non-target species may account with high frequency in a chestnut orchard [26,31]. In the present study, the presence of non-target species was quite low and *C. ilipulana* and *C. nigricana* were the most recorded species. Even if very little information is available in the literature, these non-target species are not reported as chestnut pests. They are simply attracted by the same pheromone compounds, being the geometric isomers (E,E)-, (E,Z)-, (Z,E)- and (Z,Z)-8,10-dodecadien-l-yl acetate, identified as sex pheromone components or sex attractants in the tribes Eucosmini and Grapholitini of the tortricid subfamily Olethreutinae [34,35]. Even if their presence was found to be more limited than in previous surveys [26], their presence highlights how congeneric species may be easily confused with each other, leading to a possible misinterpretation of the presence of tortrix moths in chestnut orchards.

The data of the present study report how MD was effective in reducing male moth captures. Trap catch suppression in MD plots averaged 89.5% and 93.8% for *C. fagiglandana* and 57.4% and 81% for *C. splendana* in 2019 and 2020, respectively. These results are consistent with the literature [36,37]. Ecodian^®^ CT allowed a sufficient pheromone concentration in the field for more than ten weeks of field exposure, allowing a reduction of population density (as indicated by trap catches) of both tortrix moths. Even later in the season, *C. splendana* male catches were also significantly reduced in MD plots even if the residual pheromone content in the thread was less than 10% of the initial loading. The pheromone release rate tested in Piedmont was close to previous experiences carried out in other northern and southern regions, highlighting a release duration up to 80 days with a similar trend in different environmental conditions. In almost all sites and years, the average number of male adults of both *Cydia* species collected with pheromone-baited traps was significantly lower where MD was applied compared to control plots. Only in Villar Focchiardo in 2020 was the *C. splendana* population higher in MD plots (25 individuals) when compared to control (15 individuals), even if no statistical difference was recorded.

Villar Focchiardo is the only site where trap catches in treated and control plots did not statistically differ for *C. fagiglandana* in 2019 and for *C. splendana* in 2020. Trap catches were quite small but in line with the observations carried out in the years prior to this investigation, highlighting how populations were similar during the years in all plots. No parasitism or infection was ever recorded affecting the population level.

Moreover, even if the number of males caught in traps was lower in MD plots than in the control (considering all sites and years), in Carro many *C. splendana* moths were recorded in both plots in 2019. In fact, when choosing an experimental site, topography (slope), wind exposure and size and structure of the canopy need to be carefully considered since they can affect the effectiveness of control strategies applied [38,39]. Among the surveyed sites, Carro was the windiest and therefore this abiotic condition may have affected the maintenance of an adequate pheromone coverage in the field. Furthermore, even environmental factors, such as the occurrence of wild host plants, may affect population density, as reported by Lo Verde et al. [22] for the plum fruit moth, *Grapholita funebrana* Treitschke (Lepidoptera: Tortricidae). Conversely to monophagous species, oligophagous ones, in fact, may be generally difficult to control with MD treatments because they can lay eggs on other secondary host plants [5], such as oak and beech in the case of chestnut tortrix moths [23].

The efficacy of the MD method is also related to the phenology and mating activity of the investigated species, making the placement of pheromone dispensers within the canopy a major factor for MD success. Since for both populations pupation occurs in the soil, pheromones should be deployed from the ground up to the upper part of the canopy just prior to the start of moth emergence, to reduce the incidence of mating. In the literature, applications of innovative pheromone-dispensing methods (e.g., puffers) placed in the upper part of the canopy (about 8 m in height) have been tested in chestnut orchards. However, their effectiveness has not always proved satisfactory and, therefore, further studies would be needed to optimize the pheromone doses emitted, the positioning of the device and to assess the release of pheromones in the environment [24,40]. Conversely, Ecodian^®^ CT dispensers applied vertically in the form of a wire from 0 m to 5–6 m may optimize the efficacy of this approach. The possibility of creating plumes of pheromones (conversely to the pheromone cloud typical of traditional MD through puffers or high dosage dispensers) in the flight range of the moths, makes this device more competitive on the market.

Several investigations highlighted how immigration of gravid females into pheromone-treated areas from the surroundings may represent a common obstacle when using the MD technique [40,41], but can be avoided by extending pheromone application to a larger spatial scale. Various factors contributing to border infestations, including the immigration of mated females from adjacent untreated orchards and/or reduced concentration of pheromone in the border zone, have been addressed [27,28]. In several investigations, increasing the density of dispensers in border areas and/or spraying the borders with chemicals is recommended to reduce border infestation, especially in cases of highly mobile species [5]. In the present study, to overcome this risk of invasion from neighboring areas, wires were applied on all trees surrounding the chestnut orchards within 10 m outside the perimeter of the plot. Furthermore, potential treatment interference between untreated and treated plots can occur, thus requiring that untreated and treatment areas be separated, usually by some distance. In the principles of efficacy evaluation for MD pheromones by EPPO [42], the extent of the separation of plots is reported to strictly depend on flight behavior, flight distance and prevailing wind direction, being that a larger treated area usually related to more effectiveness of the applied technique [42]. In our study, the MD plots were all 1 ha in size and separated from the control plots by at least 2 km to achieve reliable performance.

In the literature, MD is reported to work best in areas treated every year than those treated every other year. The results are in agreement with the findings by Mazomenos [41], who reported that, in MD trials, the reduction of moth populations is gradual and requires 2–3 years of continuous pheromone application to achieve control measures close to economically acceptable levels [43]. In our research, given the continuous presence of chestnut trees in the investigated sites, and the high probability of female immigration, area-wide management schemes and district strategies need to be adopted by chestnut growers to perform control efforts and achieve pest control on a large scale.

Population density may deeply affect mating disruption programs [9,28]. In the literature, this technique has been assumed to be most effective against low-density populations. Conversely, at high population densities, males can more easily locate females using visual cues as well as by chance encounters [14]. However, reduction in trap catches does not always correspond to a reduction in mating success. Onufrieva et al. [9] highlighted how MD programs targeted to control the gypsy moth can be definitely used at higher densities than ones previously considered in management programs. So far, no investigations have specifically quantified the threshold of chestnut tortrix moths above which MD treatment is no longer effective. In this study, the average number of *Cydia* collected in the surveyed sites (over 60 male adults/trap in control plot for *C. splendana* in Carro) may have negatively affected MD treatments. Thus, specific investigations on appropriate population density are needed and have to be strictly combined to background population density data to have a reliable indicator and avoid MD failures.

Concerning chestnut fruits, most *Cydia* larvae emerged in the two months following the harvest. The infestation rate recorded at the end of the storage period (late December), in all sites and years, increased on average 2 times from that observed immediately following collection, with a maximum increase of 3.8 times recorded in Badia del Borgo in 2019. Even if in a few sites the total larval damage recorded was lower for MD plots, no statistical differences were recorded when comparing MD and control plots. Only in Montese, in 2019, the larval infestation accounted for 3.2% and 10.9% in MD and control plots, respectively, corresponding to a reduction of about 71% in larval infestation. Although our results were not consistent among sites, the data recorded in Emilia-Romagna are in line with previous investigations reporting a reduction in fruit damage of 65% [36].

## 5. Conclusions

Pheromones have an important role for both monitoring and controlling insect pests. *Cydia* spp. tortrix moths have always represented a serious threat for chestnut growers, highlighting the need to develop effective environmentally friendly control strategies for their management. MD clearly proved to be effective in reducing the male population density up to 94% and 81% for *C. fagiglandana* and *C. splendana,* respectively. Regarding the release rate of the pheromone blend, current research is focusing on extending the release duration for a period exceeding 80 days (Rama F and Mereghetti P, personal communication) to avoid the risk of a late oviposition, mainly due to *C. splendana,* but also in relation to climate and phenology change.

Based on current knowledge, different methods to reduce moth populations can be used concurrently with pheromone deployment, thus refraining from chemical use. The integration of MD with the application of biocontrol methods applied in the soil (e.g., entomopathogenic fungi and nematodes), affecting overwintering larvae, may improve the efficacy of this technique. Furthermore, more research is needed to investigate if the litter management and the harvesting practices may affect their survival in the soil. Recent investigations have pointed out how the removal or conservation of organic residual (burrs, leaves, pruned materials) may affect soil properties and influence various phytosanitary threats [44,45]. That is why further studies need to be carried out to assess the role of the agroecosystem performance and resilience, evaluating pros and cons for the management of insect pests.

This study pointed out how the low catches in MD plots turned out to be a good measure of the effectiveness of communication disruption, but no satisfactory data have been obtained regarding the suppression of fruit damage in most sites. Literature data evaluating this correlation are still scant and, in many cases, provide poorly comparable results. The data obtained show that the reduction in trap catches was clearly higher than the reduction in larval damage, highlighting how the reduction in the season-long trap catches cannot be always considered as a reliable indicator of successful control. A similar experience, where pheromone was effective in reducing trap attractiveness to males without inhibiting insect mating and oviposition at the same extent, was reported for *G. funebrana* [22]. In the literature, several studies on the gypsy moth have provided a comprehensive link between the male moth trap catch and female mating success [9,14], but such a kind of study on chestnut tortrix moths, as far as necessary and desirable, is not yet available. In this respect, although the results are encouraging, changes in the sampling method (e.g., increase the sample to the whole production of individual chestnut trees) and more in-depth studies on dispersal and mating/oviposition behavior of the pests are still needed and should be encouraged.

## Figures and Tables

**Table 1:** Total number of chestnut fruits collected per site and region in MD and control plots, and percentage of damage recorded at collection and after storage in the two-year period 2019–2020. The mean proportions followed by different letters in a row are significantly different (Tukey’s test, *p* < 0.05). Damage refers to any emergence hole with a typical diameter attributable to *Cydia* spp.

2019							
Site	Region	Mating Disruption			Control		
		Total No. Chestnut Fruit	Damage (%) at Collection	Damage (%) after Storage	Total No. Chestnut Fruit	Damage (%) at Collection	Damage (%) after Storage
Villar Focchiardo	Piedmont	1617	7.80	10.64 a	1593	4.71	9.42 a
Montese	Emilia-Romagna	990	1.92	3.20 a	898	6.68	10.90 b
Badia del Borgo	Tuscany	969	2.68	8.88 a	1156	2.42	9.08 a
**2020**							
**Site**	**Region**	**Mating Disruption**			**Control**		
		**Total No. Chestnut Fruit**	**Damage (%) at Collection**	**Damage (%) after Storage**	**Total No. Chestnut Fruit**	**Damage (%) at Collection**	**Damage (%) after Storage**
Villar Focchiardo	Piedmont	1608	5.80	8.17 a	1463	5.40	8.13 a
Montese	Emilia-Romagna	926	2.27	6.70 a	986	3.45	7.00 a

## Supplementary Materials

The following are available online at www.mdpi.com/2075-4450/12/10/905/s1, Table S1: Sampling sites monitored in the two-year period 2019–2020. Figure S1: Ecodian^®^ CT pheromone dispenser applied in chestnut orchard.
