# Supplementary material for: Pheromone-Mediated Mating Disruption as Management Option for Cydia spp. in Chestnut Orchard
Source: Insects. 2021 Oct 5;12(10):905. doi: 10.3390/insects12100905 (PMC8537153; doi:10.3390/insects12100905)
Supplement: Supplementary file 1 [file insects-12-00905-s001.zip › insects-1392311-supplementary.pdf]

Supplementary Table S1. Sampling sites monitored in the two-year period 2019–2020.

Site	Province	Region	Control			Mating disruption		
			Geographic coordinates		Altitude (a.s.l.)	Geographic coordinates		Altitude (a.s.l.)
			N	E		N	E	
Villar Focchiardo	Torino	Piedmont	45°11'24.7"	07°22'57.1"	450 m	45°06'00.7"	7°14'46.2"	470 m
Carro	La Spezia	Liguria	44°06'00.6"	09°46'13.3"	413 m	44°16’16.3’’	9°37’20.5’’	421 m
Montese	Modena	Emilia-Romagna	44°19'06.1"	07°33'18.3"	857 m	44°59'16.95"	10°44'53.35"	869 m
Badia del Borgo	Firenze	Tuscany	44°04'52.5"	11°35'19.7"	331 m	44°04'30.34"	11°37'59.67"	306 m

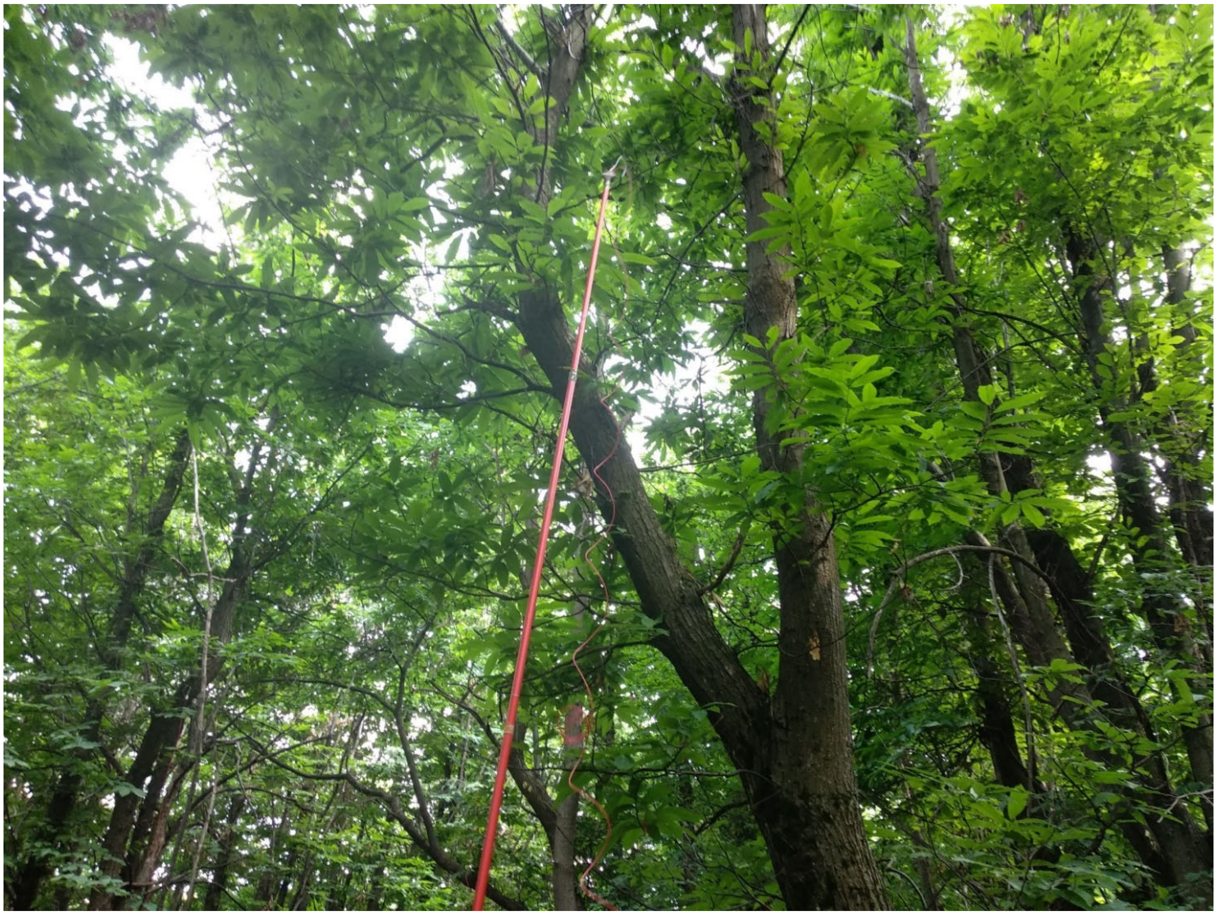

Supplementary Figure S1. Positioning of Ecodian CT® pheromone dispenser (red wire) in chestnut orchard, with the aid of lopping shears.
